# Long-term treatment outcomes of temozolomide-based chemoradiation in patients with adult-type diffuse IDH-mutant grade 2 astrocytoma

**DOI:** 10.1007/s11060-023-04418-z

**Published:** 2023-09-04

**Authors:** Giuseppe Minniti, Sergio Paolini, Manila Antonelli, Francesca Gianno, Paolo Tini, Gaetano Lanzetta, Antonella Arcella, Raffaella De Pietro, Martina Giraffa, Luca Capone, Andrea Romano, Alessandro Bozzao, Vincenzo Esposito

**Affiliations:** 1 Department of Radiological Science, Oncology and Anatomical Pathology, Umberto I Hospital, University Sapienza, Policlinico Umberto I, Rome, Italy; 2 IRCCS Neuromed, Pozzilli, IS Italy; 3 Department of Neuroscience, Sapienza University, Rome, Italy; 4 Department of Medicine, Surgery and Neurosciences, University of Siena, Siena, Italy; 5 UPMC Hillman Cancer Center, San Pietro Hospital FBF, Rome, Italy; 6 Neuroradiology Unit, NESMOS Department, Sant’Andrea Hospital, La Sapienza University, Rome, Italy

**Keywords:** IDH-mutant adult diffuse glioma, Radiotherapy, Temozolomide, Molecular markers, Grade 2 astrocytoma

## Abstract

**Purpose:**

To report the long-term outcomes in adult patients with grade 2 IDH-mutant astrocytoma treated with temozolomide (TMZ)-based chemoradiation. Methods: One hundred and three patients with histologically proven grade 2 astrocytoma received radiation therapy (RT), 50.4–54 Gy in 1.8 Gy fractions, and adjuvant TMZ up to 12 cycles. Fifty-two patients received RT at the time of tumor progression and 51 in the early postoperative period for the presence of at least one high-risk feature (age > 40 years, preoperative tumor size > 5 cm, large postoperative residual tumor, tumor crossing the midline, or presence of neurological symptoms). Overall survival (OS) and progression-free survival (PFS) were calculated from the time of diagnosis.

**Results:**

With a median follow-up time of 9.0 years (range, 1.3–15 years), median PFS and OS times were 9 years (95%CI, 6.6–10.3) and 11.8 years (95%CI, 9.3–13.4), respectively. Median PFS was 10.6 years in the early treatment group and 6 years in delayed treatment group (hazard ratio (HR) 0.30; 95%CI 0.16–0.59; *p* = 0.0005); however, OS was not significantly different between groups (12.8 vs. 10.4 years; HR 0.64; 95%CI 0.33–1.25; *p* = 0.23). Extent of resection, KPS, and small residual disease were associated with OS, with postoperative tumor ≤ 1 cc that emerged as the strongest independent predictor (HR: 0.27; 95%CI 0.08–0.87; *p* = 0.01).

**Conclusions:**

TMZ-based chemoradiation is associated with survival benefit in patients with grade 2 IDH-mutant astrocytoma. For this group of patients, chemoradiation can be deferred until time of progression in younger patients receiving extensive resection, while early treatment should be recommended in high-risk patients.

## Introduction

The fifth edition of the World Health Organization (WHO) Classification of Tumors of the Central Nervous System (CNS) published in 2021 classifies adult-type diffuse gliomas into IDH-mutant astrocytoma, IDH-mutant and 1p/19q-codeleted oligodendroglioma, and IDH-wildtype glioblastoma [1]. IDH-mutant astrocytomas are characterized by the presence of IDH mutations, frequent ATRX and/or TP53 mutation, and absence of 1p/19q codeletion. Grade 2 and 3 astrocytomas present the same molecular profile, although grade 2 tumors are well-differentiated, low cellular, slow-growing tumors [2]. The growth fraction as determined by the Ki-67 proliferation index is usually < 4%, mitotic figures are rare, and microvascular proliferation and necrosis are absent.

Two Radiation Therapy Oncology Group (RTOG) studies have evaluated the impact of combining chemotherapy and radiation therapy (RT) in patients with grade 2 gliomas [3–5]. In the RTOG 9802 phase III trial, 251 patients with “high-risk” low-grade glioma, defined by patients aged ≥40 years following any extent of resection or those aged 18–39 years who had received subtotal resection or biopsy, were randomized to receive RT (54 Gy in 1.8 Gy fractions) alone or RT followed by six cycles of procarbazine, CCNU, and vincristine (PCV). With a median follow-up time of 11.9 years, longer overall survival (OS) was observed for patients receiving combined chemoradiation, increasing from 7.8 years to 13.3 years [4]. As confirmed in a subsequent genomic analysis of the trial, survival benefit was observed in patients with either IDH-mutant astrocytoma or oligodendroglioma but not in those with IDH-wild-type tumor [6]. In patients with grade 2 astrocytoma, OS and progression-free survival (PFS) times were 11.4 years and 10.4 years, respectively, supporting the use of RT followed by PCV as standard treatment for high-risk low-grade patients who are deemed to require post-surgical treatment.

The RTOG 0424, single-arm, phase 2 study assessed the efficacy of RT (54 Gy in 30 fractions) with TMZ up to 12 cycles in 129 patients with low-grade glioma with three or more risk factors, including age ≥ 40 years, astrocytoma histology, bihemispheric tumor, size ≥ 6 cm, or preoperative neurologic function status > 1). At a median follow-up for surviving patients of 9.0 years, 3-year OS was 73.5% (95% confidence interval, 65.8-81.1%), numerically superior to the 3-year OS historical control of 54% observed with RT alone (*p* < 0.001) [7].

In this study we report the long-term results of RT and adjuvant TMZ given as immediate post-operative treatment or deferred at the time of tumor progression in patients with IDH-mutant grade 2 astrocytoma. In addition, the impact of prognostic factors on clinical outcomes has been evaluated.

## Patients and methods

### Patients

One hundred and twenty consecutive adult patients with histologically proven IDH-mutant grade 2 astrocytoma according to the 2021 WHO classification, with Karnofsky performance status (KPS) score greater than or equal to 60, who received RT followed by adjuvant TMZ in four Italian Institutions between October 2004 and March 2020 were evaluated. Patients received RT in the early postoperative period or at the time of tumor progression followed by 12 cycles of TMZ. Early post-operative treatment was administered in presence of at least one high-risk feature, defined as age > 40 years, preoperative tumor size > 5 cm, large postoperative residual tumor, tumor crossing the midline, or presence of neurological symptoms. All clinically relevant radiographic, surgical, and pathologic information were drawn from a prospectively maintained database of patients with brain tumors treated at IRCCS Neuromed and UPMC San Pietro FBF, or from medical charts for those treated at Sapienza University Policlinico Umberto 1 and Sant’Andrea medical hospitals. After exclusion of 17 patients due to insufficient molecular and clinical information (n = 12) or previous RT treatment (n = 5), a total of 103 patients remained in the final analysis. All patients gave their consent to the treatment. The local Institutional Review Boards approved this retrospective study.

RT consisted of fractionated focal irradiation using a dose of 50.4–54 Gy delivered in 28–30 daily fractions of 1.8 Gy each. In patients receiving early postoperative treatment, RT started within twelve weeks after surgery. Eight-nine patients received intensity-modulated radiation therapy (IMRT) or volumetric modulated arc therapy (VMAT), while 14 patients received 3D-conformal RT. The gross tumor volume (GTV) was defined as the surgical cavity and visible residual disease on the postoperative magnetic resonance imaging (MRI) T2/FLAIR sequences. The clinical target volume (CTV), which encompasses all areas of potential microscopic tumor infiltration, was defined as the GTV plus 1 cm margin reduced at natural anatomical barriers (e.g. tentorium, skull, ventricles); then, a 3 mm safety margin expansion in all directions was added to the CTV to generate the planning target volume (PTV) to account for daily setup variation. Adjuvant TMZ was generally started within 6 weeks after the end of RT and delivered for 5 days every 28 days up to 12 cycles. The dose was 150 mg/m^2^ for the first cycle and was increased to 200 mg/m^2^ from the second cycle. The dose was reduced or suspended for patients who developed grade 3 or 4 toxicity according to the NCI Common Terminology Criteria for Adverse Events (CTCAE v.5.0), or with disease progression [8].

All patients were evaluated clinically before RT treatment and monthly during adjuvant TMZ, and every 6 months thereafter. MRI was repeated before and 4 weeks after RT, every 3 months during adjuvant TMZ, and thereafter every 6 months, or as appropriate. Neuroradiographic response was assessed by the Response Assessment in Neuro-Oncology (RANO) [9]. In brief, progressive disease (PD) was defined as significant increase in T2/FLAIR lesions on stable or increasing doses of corticosteroids, or as any new lesion. Complete response was defined as complete disappearance of all enhancing measurable and non-enhancing non-measureable (T2/FLAIR) lesions, partial response as decrease of non-enhancing lesions ≥ 50%, minor response as decrease of non-enhancing lesions of 25–50%, and stable disease as any other condition. Responses were reviewed by the same neuroradiologists (A.B and A.R). Transient radiological abnormalities characterized by increased edema and contrast-enhancement on MRI, which occurred within 12 weeks from the end of RT and stabilized or resolved on subsequent clinical and radiographic assessments without a change in therapy, were recorded as pseudoprogression.

### Laboratory methods

Pathology review, immunohistochemistry (IHC) and molecular testing were performed centrally in the same laboratory (FG, M.A, A.A). IHC staining was performed automatically using an automatic immunostaining Ventana® Benchmark® XT/ULTRA. Tissue sections were cut at 5 μm, dried at 70 °C for 30 min. and then dewaxed. Pretreatment with heat-induced antigen retrieval was performed prior to IHC analysis. Sections were incubated for 30 min with ATRX (NBP1-83077, rabbit polyclonal, 1:1000 dilution; Novus Biologicals), p53 (NCL-L-p53-DO7, mouse polyclonal, 0.875 µg/mL; Leica Novocastra) and IDH1 R132H (H09, mouse monoclonal, 1:100; Dianova) for canonical IDH1 mutation. To assess for noncanonical IDH1/2 mutations (not recognized by IHC), DNA sequencing was performed. The DNA extracted from the tissue tumors containing at least 70% of neoplastic cells, using ReliaPrep FFPE gDNA Miniprep System (Promega), was amplified by polymerase chain reaction (PCR) with a pair of specific primers for exon 4 of IDH1/2 genes. *TERTp* mutations were analyzed via pyrosequencing: PCR primers were designed to amplify the TERTp region containing the C228T and C250T hotspots. Loss of heterozygosity (LOH) on chromosomes 1p and 19q, was analyzed by PCR using two different pairs of fluorescent primers for each chromosome. Fluorescentdye-labeled PCR products of the microsatellite marker loci were analyzed on an ABI PRISM 377 automated DNA Sequencer (Applied Biosystems). For the *MGMT* promoter methylation analysis, bisulfite modification was performed on 100 ng of genomic DNA using the EpiTech Bisulfite kit (Qiagen) according to the manufacturer’s instructions.

### Statistical analysis

OS was estimated using the Kaplan-Meier method calculated from the time of diagnosis. PFS was calculated using a cumulative incidence function, with death without progression treated as the competing risk. Patients without an event were censored at the last contact. Chi-squared and nonparametric Mann-Whitney tests were used to examine between-group covariate differences, and the Cox proportional hazards model was used for univariate and multivariate analysis to assess the effects of clinical/treatment variables on clinical outcomes. The variables with unadjusted P-value < 0.1 in univariate analyses were considered confounders and included in the multivariate Cox model to identify independent prognostic factors. Multicollinearity among explanatory variables was analyzed using the variance inflation factor, with values < 3 being considered acceptable. Results were given as hazard ratios (HR) with 95% confidence intervals (CI). All the significance tests were two-tailed. The threshold for statistical significance was set at a p-value < 0.05. Statistical evaluation was performed using a commercial statistical software package (StatView, version 5.0, SAS Institute, Cary, NC).

## Results

One hundred and three consecutive patients (52 males and 51 females) with IDH-mutant grade 2 astrocytoma who underwent RT plus TMZ between February 2005 and March 2020 were analyzed. Pre-treatment characteristics of patients are listed in Table 1. The median age was 39 years (range 20–68 years) and median KPS was 100 (range, 60 to 100). Proportion of resection defined by preoperative volume - postoperative volume/ preoperative volume was based on preoperative and immediate postoperative volumetric T2/FLAIR MRI [10]. Thirteen (12.5%) patients were considered to have macroscopically complete resection (100% extent of resection), 36 (35%) near total subtotal resection (> 90% and ≤ 5 cm^3^ residual tumor volume), 31 (30%) subtotal resection (≥40% and ≤ 25 cm^3^ residual tumor), and 19 (18.5%) partial resection (1–39% ± residual tumor > 25 cm^3^). Four patients underwent stereotactic biopsy. Amongst 94 patients with good quality data to assess the MGMT promoter methylation status, 90 (95.7%) had a methylated tumor, and 4 (4.3%) an unmethylated tumor. Fifty-one (49.5%) patients received early postoperative treatment and 52 (50.5%) patients received deferred treatment at the time of disease progression. Clinical characteristics of two groups were similar; however, postoperative residual volumes were significantly larger in the early RT treatment group (Table 1). For patients who received early postoperative chemoradiation, the distribution of high-risk factors was 34%, 34%, 24%, and 8% for 2,3,4, or 5 risk factors. In  21 patients who underwent subtotal (n = 16) or partial resection (n = 5)  the treatment was deferred beacuse t they were younger than 34 years of age, had a KPS score of 100% and relatively little postoperative residual tumor (9-26.4 ml). A second surgery was performed on 14 patients who received delayed RT; histology confirmed grade 2 astrocytoma for all tumors. At the time of analysis (April 2023), 47 (45.6%) patients had progressed and 37 (35.9%) patients had died, most (95%) of brain tumor.

**Table 1:** Baseline characteristics of patients

Characteristics	All (n = 103)	Early treatment (n = 50)	Delayed treatment (n = 53)	p-value
**Age (years)**				0.7
Median	39	40	38	
Range	19–68	23–68	20–63	
**Sex**				0.9
Male	52	26	26	
Female	51	24	27	
**Karnofsky performance status**				0.7
Median	100	90	100	
Range	60–100	60–100	70–100	
**Site of tumor**				0.7
Temporal	32	15	17	
Frontal	52	28	24	
Parietal	11	5	6	
Occipital	8	2	6	
**RTOG Neurologic Function Status**				0.6
0	54	25	29	
1	33	15	18	
2	13	8	5	
3	3	2	1	
**Radiation dose**				0.6
54 Gy/30 fractions	62	32	30	
50.4 Gy/28 fractions	41	18	23	
**Extension of resection**				0.3
Total	13	4	9	
Near total	36	13	23	
Subtotal	31	15	16	
Partial/biopsy	23	18	5	
**MGMT promoter methylation status**				1.0
Methylated	91	46	45	
Unmethylated	3	1	2	
Unknown	9	3	6	
**Postoperative residual volume (cm3)**				
Median	24.2	22.5	4.5	0.001
Range	0-251.3	0-251.3	0-26.4	
**Target volumes (cm3)**				
**Gross tumor volume (GTV)**				
Median	56.8	49.24	68.9	0.02
Range	11.4-251.3	11.4-251.3	29.2-113.4	
**Clinical tumor volume (CTV)**				
Median	166.7	164.4	176.8	0.15
Range	72.3-437.5	72.3-437.5	87.5-348.2	
**Planning tumor volume (PTV)**				
Median	226.5	211.2	234.2	0.2
Range	102.1-541.3	102.1-541.3	94.6-437.3	

### PFS and OS analysis

**Fig. 1:**
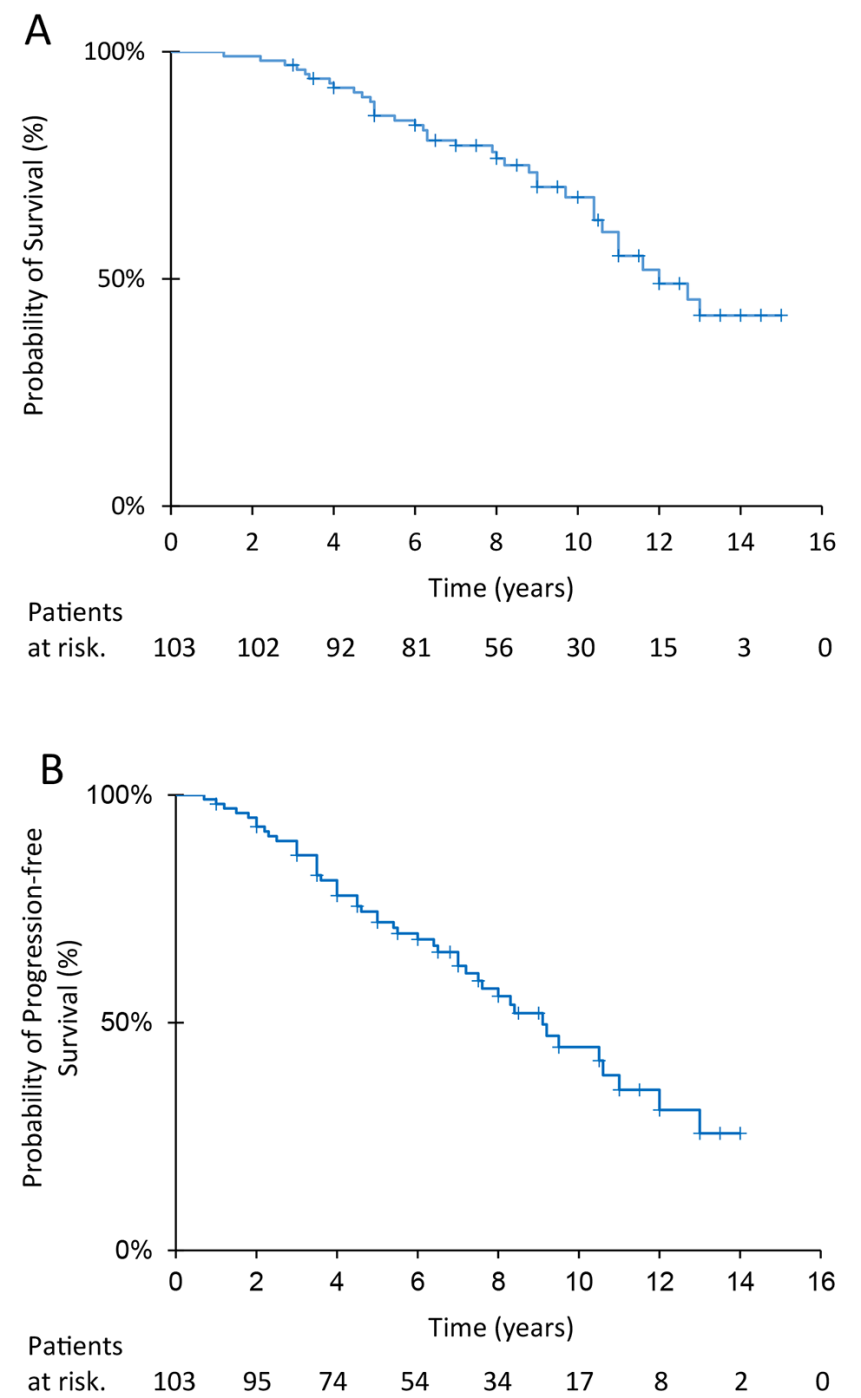
Survival and progression-free survival of patients treated with radiation therapy and temozolomide

With a median follow-up time of 9.0 years (range, 1.3–15 years), median PFS and OS times were 9 years (95%CI, 6.6–10.3) and 11.8 years (95%CI, 9.3–13.4), respectively (Fig. 1). Five-year and 10-year survival rates were 86% (95%CI, 79.3–92.8) and 67% (95%CI, 56.3–77.5), respectively; 5-year and 10-year PFS rates were 71.3% (95%CI, 58.1–80.5) and 42% (95%CI, 26.3–53.7), respectively.

**Fig. 2:**
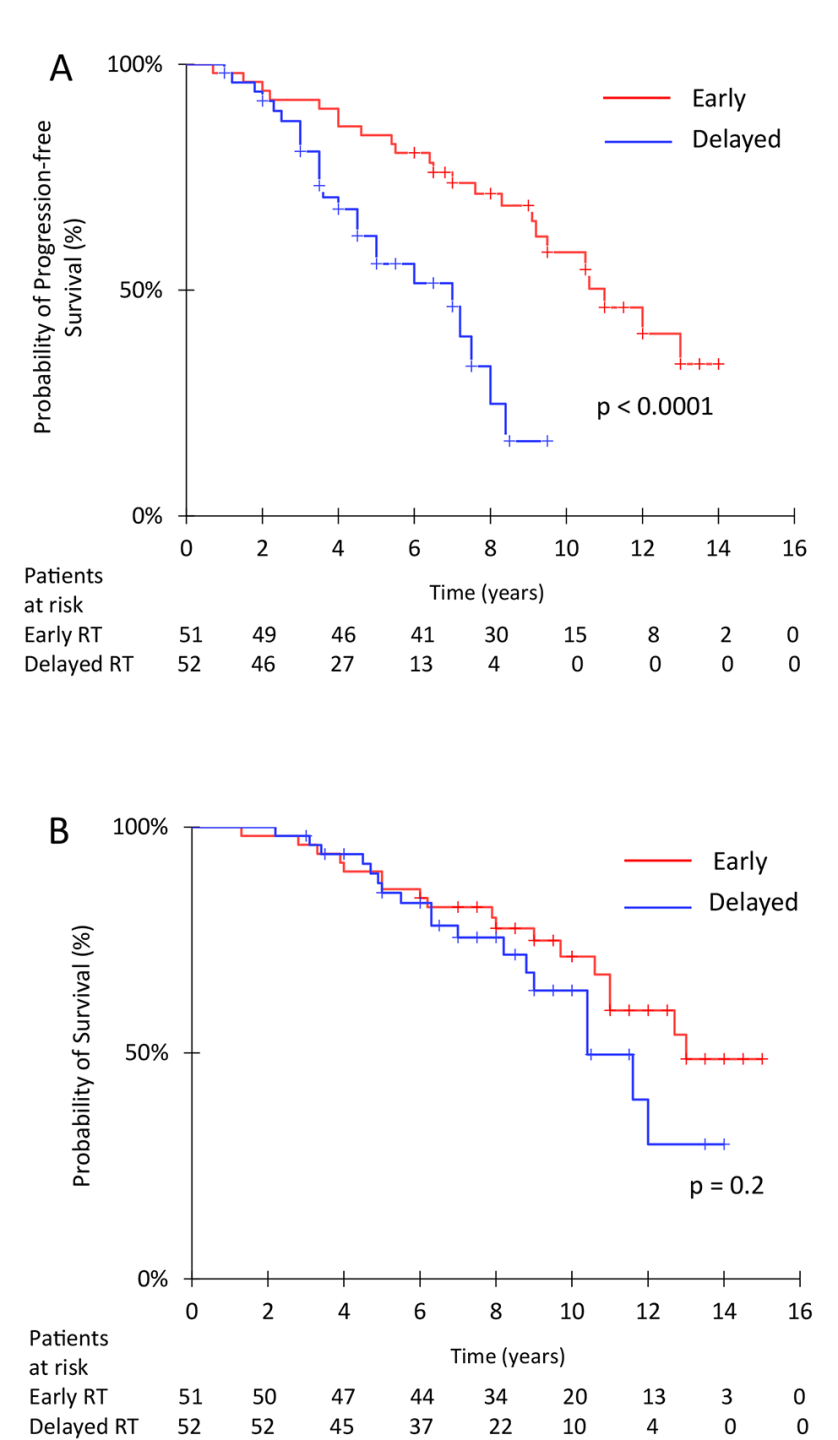
Progression-free survival and survival for patients receiving early treatment or deferred treatment until the time of progression. No significant survival differences were observed between groups, but early radiation therapy and temozolomide significantly improved progression-free survival

OS was not significantly different between patients receiving early postoperative irradiation and those who had delayed irradiation (Fig. 2, panel A). Median survival times were 12.8 years in early treated group and 10.4 years in delayed treatment group (HR: 0.64; 95%CI 0.33–1.25; *p* = 0.23); respective 5-year and 10-year survival rates were 86.3% (95%CI, 76.8–95.7) and 71.4% (95%CI, 55.7–85.0), and 85.8% (95%CI, 76-95.6) and 61.2% (95%CI, 44.7–77.7). Median PFS was significantly better in early postoperative irradiation group: 10.6 years in the early RT group and 6.0 years in delayed RT group (HR: 0.30, 95%CI 0.16–0.59; *p* = 0.0005) (Fig. 2, panel B). Five-year and 10-year PFS rates were 82.6% (95%CI, 71.9–92.8) and 58% (95%CI, 41.7–75.3) in the early RT group and 54.6% (95%CI, 38.9–70.2) and 18.5% (95%CI, 0.3–36.7) in the delayed RT group. A complete response was achieved in 4 patients, a partial response in 16 patients, and a minor response in 22 patients.

**Fig .3:**
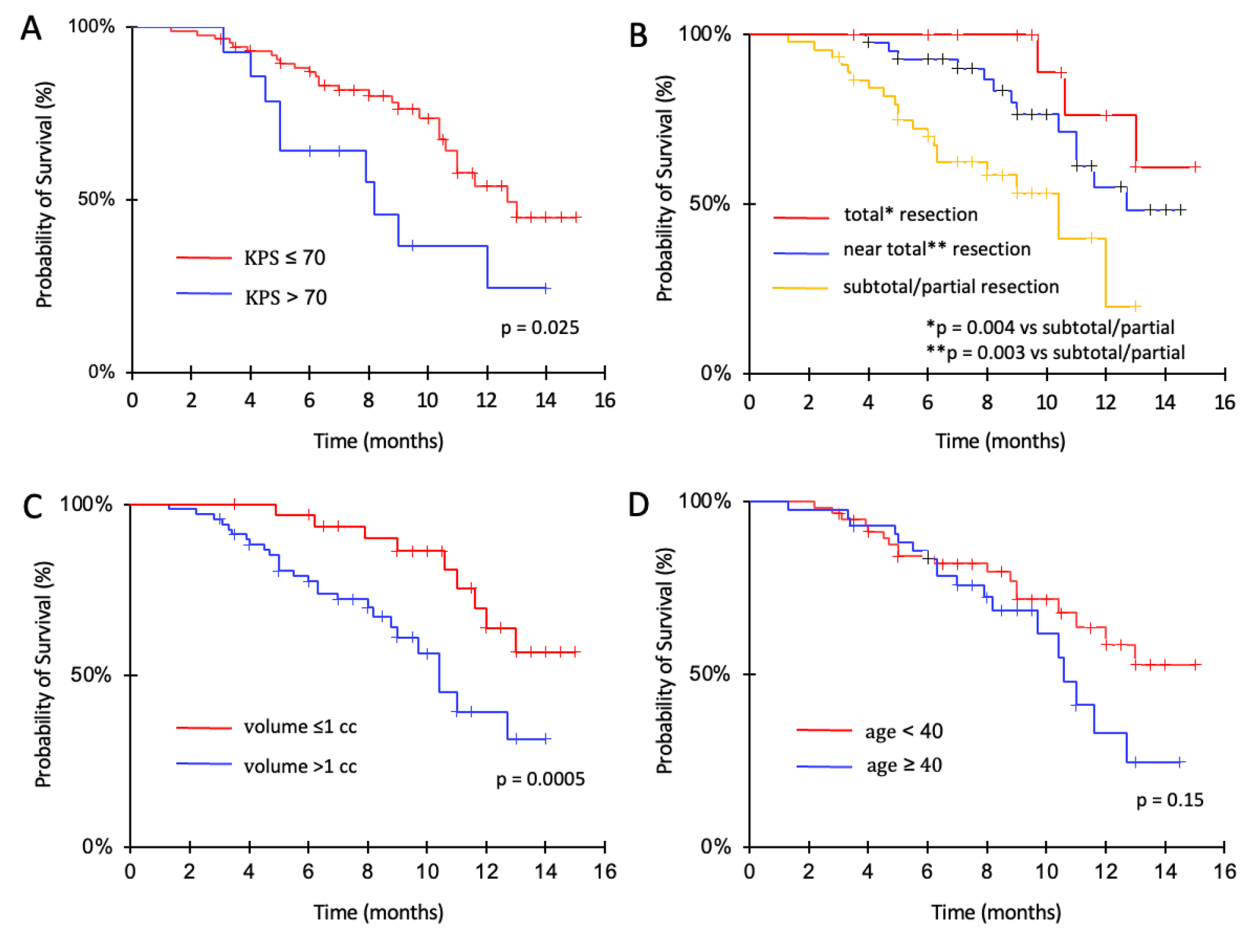
Survival according to KPS (**A**), extent of resection (**B**), residual tumor volume (**C**), and age (**D**). Patients receiving total or near total resection displayed significantly longer survival than those with subtotal/partial resection (p=0.0037 and p=0.0025). Likewise, patients with KPS > 70 (p= 0.025) and postoperative residual tumor volume ≤ 1cc (p=0.0005) presented better survival. In contrast, age did not impact survival

At univariate analysis, extent of resection (total vs. subtotal/partial resection; HR: 0.30; 95%CI 0.04–0.653; *p* = 0.0037; near total vs. subtotal/partial resection; HR: 0.22; 95%CI 0.09–0.58; *p* = 0.0025), higher KPS score (KPS > 70 vs. KPS ≤ 70; HR: 0.42; 95%CI 0.21–0.87; *p* = 0.025), and minimal residual disease (≤ 5 cc vs. > 5 cc; HR: 0.36; 95%CI 0.17–0.76; *p* = 0.009; ≤1 cc vs. > 1 cc; HR: 0.18; 95%CI 0.07–0.47; *p* = 0.0005) were associated with longer OS (Fig. 3). Age (< 40 vs. ≥40 years), site of disease, treatment volume, MGMT methylation status, RT techniques and dose did not affect survival; however, smaller treatment volume were of borderline significance (*p* = 0.088). At multivariate analysis, minimal residual disease was associated with longer survival with volume ≤ 1 cc as the strongest independent predictor (HR: 0.27; 95%CI 0.08–0.87; *p* = 0.01). No factors affected PFS other than treatment timing. Total vs. subtotal/partial resection was of borderline significance (HR: 0.41; 95%CI 0.06–1.06; *p* = 0.063).

Patients who progressed after chemoradiation were treated by one or more second-line chemotherapy regimens (67%), surgery (24%), and a second course of RT (16%) given alone or in combination. Chemotherapy was lomustine in 16 (15.5%) patients, fotemustine in 8 (7.7%) patients, temozolomide re-challenge in 47 (45.6%) patients, and bevacizumab in 11 (10.6%) patients.

### Toxicity

All patients completed the planned programme of RT. During adjuvant TMZ, 21 (20.3%) patients had grade 3/4 thrombocytopenia, 12 (11.6%) grade 3/4 neutropenia, 8 (7.7%) grade 3/4 anemia, and 39 (37.8%) grade 3/4 lymphocytopenia. TMZ was stopped in 7 (6.8%) patients and delayed or reduced in 16 (15.5%) patients. A grade 2 or 3 fatigue occurred in 22 (25%) patients and grade 2 or 3 nausea in 22 (25%) patients. Two patients had a severe lung infection (pneumonia) that resolved with medical therapy, and one patient had a deep vein thrombosis.

MRI imaging abnormalities characterized by increased edema and contrast enhancement which stabilized or resolved on subsequent clinical and radiographic follow-up were observed in 17 patients and were recorded as pseudoprogression. Amongst them, a transient neurological deterioration occurred in 9 patients, requiring medical treatment with corticosteroids.

## Discussion

In this multicenter study, where patients with grade 2 IDH-mutant astrocytoma received RT and TMZ, median PFS and OS times were 9 years and 11.8 years, respectively. Immediate postoperative chemoradiation increased PFS by 4 years compared with delayed treatment; however, increased PFS did not translate into OS benefit. With a median follow-up of 9.0 years, median survival times and 10-year survival rates were 12.8 years and 71.4% in the early treatment group, respectively, and 10.4 years and 61.2% in the delayed treatment group, respectively.

The survival time observed in our study with TMZ-based chemoradiation is consistent with those reported in secondary analysis of previous studies using RT in combination with PCV or TMZ and compares favorably with results observed following RT or chemotherapy alone [3–7, 11]. Long-term results of the randomized RTOG 9802 trial demonstrated a significant survival benefit with the addition of adjuvant PCV to RT in patients with IDH-mutant tumors, both co-deleted and non-co-deleted, but not in those with IDH-wild-type tumors [4]. For astrocytoma subgroup, the median OS was 11.4 years for 22 patients treated with chemoradiation and 4.3 years for 21 patients receiving RT alone (HR 0.38; *p* = 0.013) [6]. In the single-arm, phase 2 RTOG 0424 study of patients with either IDH-mutant or IDH-wild-type “high-risk” low-grade glioma receiving RT plus concurrent and adjuvant TMZ, median survival time of 8.2 years and 10-year survival rate of 34.6% were superior to those observed in historical controls treated with RT alone [5]. A recent analysis of the trial including 80 patients with complete molecular data showed that IDH-mutant tumors were associated with significantly better survival compared with IDH-wild-type tumors; median OS and PFS times were 9.4 and 8.1 years in the oligodendroglioma group, 8.8 and 7.5 years in the astrocytoma group, and 2.3 and 1.0 years in the IDH-wild-type group, respectively [12]. The use of chemotherapy alone as frontline therapy might be an option if RT is not feasible. In the EORTC 22,033–26,033 phase III study, 477 patients with a low-grade glioma with at least one high-risk feature were randomized to either conformal RT, 50.4 Gy/28 fractions, or dose-dense TMZ [11]. In patients with grade 2 astrocytoma, PFS was 55.3 after RT and 36 months after TMZ At a median follow-up of 48 months (*p* = 0.004). Overall, available data support the use of combined chemoradiation as standard of care for patients with WHO grade 2 astrocytoma deemed to require post-surgical treatment. Whether PCV is superior to TMZ remains an open question that needs to be evaluated in future trials.

Timing of the treatment remains a controversial issue. In the EORTC 22,845 trial, where 354 adult patients with either low-grade astrocytoma or oligodendroglioma were randomly assigned to receive early RT or deferred RT until the time of progression, an early treatment lengthened the period without progression but did not affect OS [13]. The timing of RT was not specifically addressed in the RTOG 9802 trial, although 5-years PFS rate of 48% observed in 116 patients with favorable low-grade glioma, those aged < 40 years of age who received gross total resection, was similar to 5-year PFS rate of 50% observed in unfavorable patients treated with postoperative RT with or without PCV chemotherapy [14]. Significant PFS benefit observed in the early treatment group could be important in patients at risk of neurological worsening, such as those with preexisting symptoms, large residual tumors, or tumors located in eloquent areas. Therefore, a close surveillance can be recommended for young patients who are asymptomatic or with seizures only after total resection; in contrast, adjuvant RT should be considered for patients “high-risk“ features [2, 15].

In our study we have investigated the impact of prognostic factors on survival. In univariate analysis, KPS score > 70, total/near-total resection, and small postoperative residual tumor ≤ 5 and ≤ 1 cc were associated with longer survival; however, only a residual tumor ≤ 1 cc was an independent prognostic factor for survival. In contrast, the use of a total dose of 54 Gy provided no additional benefit compared with lower radiation dose, confirming previous study showing no difference in clinical outcome between high-dose versus low-dose radiation in adult low-grade glioma [16, 17]. MGMT promoter methylation was present in almost all patients as previously reported confirming that MGMT testing does did not provide additional prognostic or predictive value in this population [11]. Overall, our study support maximal resection in patients with IDH-mutant grade 2 astrocytoma. Wijnenga et al. [18] showed that postoperative volume was associated with OS in 229 patients who underwent surgery for a supratentorial low grade glioma, showing that even very small postoperative volumes of 0.1 to 5.0 cm^3^ negatively affected the outcome. In our study, complete resection was also associated with longest survival, although its impact in multivariate analysis appeared limited probably due to the small number of patients with completely resected tumors.

Our study has several limitations, owing to its retrospective nature. Choice of treatment was determined by physician and patient preferences, thus introducing bias. Moreover, unmeasured baseline characteristics that may contribute to the observed differences in OS and PFS between groups. A randomized trial would be the ideal way to compare early versus delayed treatment.

In conclusion, our study indicates that TMZ-based chemoradiation is associated with survival benefit in patients with IDH-mutant grade 2 astrocytoma. An early treatment is associated with longer PFS compared with delayed treatment, but survival times are equivalent. In absence of randomized trials evaluating early versus delayed chemoradiation in this specific WHO-defined molecular subgroup, current data indicate that chemoradiation can be deferred in younger patients receiving extensive resection, while early post-operative treatment should be recommended in so defined “high-risk” patients. Whether TMZ is equivalent to PCV remains an open question that need to be addressed in future trials.
